# Oxidized β-Carotene Is a Novel Phytochemical Immune Modulator That Supports Animal Health and Performance for Antibiotic-Free Production

**DOI:** 10.3390/ani13020289

**Published:** 2023-01-14

**Authors:** William W. Riley, James G. Nickerson, Trevor J. Mogg, Graham W. Burton

**Affiliations:** 1 International School, Jinan University, Guangzhou 510632, China; 2 Avivagen, Inc., Ottawa, ON K1A 0R6, Canada

**Keywords:** antibiotic alternative, oxidized β-carotene, growth and health performance, metaphylaxis, sustainability

## Abstract

**Simple Summary:**

Oxidized β-carotene (OxBC), a phytochemical that occurs naturally in plants, including fruits and vegetables, is formed by the spontaneous reaction of β-carotene with ambient oxygen. Synthetic OxBC, obtained by the full oxidation of β-carotene with air, shows considerable promise as a parts-per-million in-feed antimicrobial alternative additive that enhances health and performance in poultry, swine, and ruminant species. OxBC is predominantly composed of β-carotene–oxygen copolymers that have beneficial immune-modulating effects. These effects occur within the innate immune system by priming it to face environmental challenges and by mitigating the inflammatory response in a timely fashion, but without any direct anti-bacterial activity. As well, OxBC modulates but does not stimulate and deplete the animal’s energy stores unless directly stress-challenged.

**Abstract:**

Oxidized β-carotene (OxBC), a phytochemical that occurs naturally in plants, is formed by the spontaneous reaction of β-carotene with ambient oxygen. Synthetic OxBC, obtained by full oxidation of β-carotene with air, shows considerable promise as an in-feed antimicrobial alternative additive that enhances health and performance in livestock. OxBC is predominantly composed of β-carotene-oxygen copolymers that have beneficial immune-modulating effects that occur within the innate immune system by priming it to face microbial challenges and by mitigating the inflammatory response. OxBC does not have any direct anti-bacterial activity. Further, unlike traditional immune stimulants, OxBC modulates but does not stimulate and utilize the animal’s energy stores unless directly stress-challenged. These immune effects occur by mechanisms distinct from the provitamin A or antioxidant pathways commonly proposed as explanations for β-carotene’s actions. Trials in poultry, swine, and dairy cows with low parts-per-million in-feed OxBC supplementation have shown performance benefits over and above those of feeds containing regular vitamin and mineral premixes. Through its ability to enhance immune function, health, and performance, OxBC has demonstrated utility not only as a viable alternative to in-feed antimicrobials but also in its ability to provide tangible health and performance benefits in applications where antimicrobial usage is precluded.

## 1. Antimicrobial Alternatives for Enhancement of Health and Performance

Increased regulatory pressure banning or limiting the use of antibiotic growth promoters (AGPs) and growing consumer demand for sustainable agriculture practices, including products ‘Raised Without Antibiotics’ or ‘No Antibiotics Ever’, have been driving the search for alternative products [1,2,3,4]. Much effort has been made to discover and develop alternatives to AGPs to maintain or improve livestock health and performance. Alternatives include probiotics, prebiotics, synbiotics, organic acids, enzymes, phytogenics, antimicrobial peptides, hyperimmune egg antibodies, bacteriophages, clays, and metals. The mechanism of action, efficacy, and advantages and disadvantages of their uses have been reviewed [4]. Although the beneficial effects of many of the alternatives have been demonstrated, the consensus is that many currently available products lack consistency, and results can vary greatly. Additionally, their modes of action often are not well understood.

Antibiotic alternatives are intended to replace AGPs, whose primary function is to decrease microbial populations and promote growth via many different modes of action that may include alteration and/or inhibition of microbial growth, decreased inflammation, enhancement of innate immunity, reduced oxidative stress, and improved gut integrity [5]. 

There is no clear consensus on how AGPs act to promote antibiotic-mediated growth enhancement, although several ideas have been proposed [4]. An attractive hypothesis proposed by Niewold is that beneficial effects occur due to the interaction of antibiotics with host immune cells rather than from growth-inhibitory effects on microbiota [6]. Niewold proposed that antibiotics lower the inflammatory response and, thus, the production of proinflammatory cytokines that reduce the appetite and promote muscle catabolism. The anti-inflammatory role of AGPs reduces wasted energy and directs it toward production. 

### 1.1. Classes of Alternatives 

In reviewing alternatives, Gadde et al. commented that ideally, an alternative should have the same beneficial effects as AGPs to ensure optimum animal performance and to increase nutrient availability [4]. Two attractive proposed mechanisms of action of AGPs are modulation of the microbiome and immune activities. A practical alternative should possess both these properties in addition to improving feed conversion or growth or, ideally, both. Of the several classes of alternatives that have been considered, immunomodulators are an attractive opportunity.

### 1.2. Immune Modulators

An important feature of immune modulators, in contrast to vaccines, for example, is their ability to affect the immune system in a way that is less dependent on a specific pathogen threat, making them effective against a broad range of pathogens [7].

### 1.3. Phytochemicals

Phytochemicals are natural bioactive compounds that are derived from plants and incorporated into animal feeds to enhance productivity [5]. In recent years, phytochemicals have been used as natural growth promoters in the ruminant, swine, and poultry production industries. A wide variety of herbs and spices (e.g., thyme, oregano, rosemary, marjoram, yarrow, garlic, ginger, green tea, black cumin, coriander, and cinnamon) have been used in poultry for their potential application as AGP alternatives.

Lillehoj et al. [5] noted that a growing body of scientific evidence has demonstrated that many of the health-promoting activities of phytochemicals are mediated through their ability to enhance host defense against microbial infections. The immune-activating properties of medicinal plants such as dandelion, mustard, and safflower have been evaluated in vitro. All three extracts inhibit tumor cell growth, stimulate innate immunity, and exert antioxidant effects in poultry.

Our research, aimed at a broader understanding of carotenoid biochemistry, led to the discovery of a new class of phytochemical compounds with immunomodulatory properties that derive from the full oxidation of β-carotene [8]. Collectively, these compounds are referred to as oxidized β-carotene (OxBC), and their natural occurrence and immunological activity shed significant new light on the carotenoid mode of action [9]. Furthermore, as reviewed in the following sections, OxBC shows significant promise as an AGP alternative.

## 2. Carotenoids. Sources of Phytochemicals with a Unique Role in Health Promotion

The role of various carotenoids, including β-carotene, as natural pigments, and precursors of the retinoids with vitamin A activity, has long been known. However, over the last three decades, considerable empirical and scientific evidence has been gathered that suggests that carotenoids and their metabolites have functions beyond those that have been characterized as “normal” physiological roles [10,11,12,13]. These new roles are considered “functional”, as in functional foods, i.e., providing benefits beyond those attributable to the nutrient value of the compounds. This generally implies a health benefit that is in addition to the documented physiological function of the nutrient or related metabolite(s). Thus, carotenoids have been implicated in the reduced risk involved in the development of chronic diseases, for example, some types of cancers and certain cardiovascular and eye diseases [13,14,15].

Carotenoids represent the most abundant lipid-soluble phytochemicals. In vitro and in vivo studies have suggested they have antioxidant, antiapoptotic, and anti-inflammatory properties, many of which have been linked to the effect of carotenoids or their metabolic or breakdown products (apocarotenoids) on intracellular signaling cascades influencing gene expression and protein translation [12].

Although beneficial health effects attributed to carotenoids frequently have been attributed to a purported antioxidant capability [16], there are other plausible mechanisms of action of carotenoids and their apocarotenoids, including immunomodulatory and anti-inflammatory activities [10,13,17,18,19].

Carotenoids have been implicated in the modulation of the avian innate immune system [20,21,22,23,24]. Koutsos et al. [25] used lipopolysaccharide (LPS) or interleukin-1 (IL-1) to induce an acute phase response in broiler chicks and concluded that this response was implicated in reducing tissue carotenoid levels during infectious disease. In a subsequent study, this same group demonstrated that chicks fed 0 mg lutein had greater body weight losses and higher plasma haptoglobin and relative thymus, bursa, and spleen weights post-LPS challenge compared with chicks fed 40 mg lutein/kg diet [26]. The conclusion was that a lack of carotenoid exposure, either in ovo or post-hatch, increased systemic inflammation and that the innate immune response is critical as the first line of defense against invading pathogens. The components of this innate immune response possess the ability to regulate the transcription and translation of genes for additional mediators of the inflammatory response (i.e., cytokines and acute-phase proteins). For example, plasma haptoglobin and serum amyloid A protein are acute-phase proteins that respond positively to inflammatory stimulation in various species, including wild birds [27,28,29].

In livestock animals it was thought for some time that β-carotene is a source of activity beyond its provitamin A status. However, in 2012 in a review of the safety and efficacy of β-carotene as a feed additive for all animal species, an EFSA panel concluded that non-provitamin A effects, for example, on reproduction and immunity, had not yet been sufficiently demonstrated [30].

Subsequently, Burton et al. expanded upon the potential role of dietary carotenoids in the modulation of the innate immune system in various animal species by describing how naturally occurring carotenoids undergo spontaneous, autocatalytic oxidation (autoxidation) to produce many complex oxygen copolymers [8]. They suggested that these copolymers are the source of immune-enhancing activity in several species, including poultry, that formerly had been attributed to intact carotenoids [9]. 

## 3. Oxidized β-Carotene (OxBC). A Naturally Occurring Phytochemical Immunomodulator

### 3.1. What Is OxBC? Oxygen Copolymer and Apocarotenoid Compounds

The autoxidation of highly unsaturated hydrocarbon compounds preferentially proceeds by the addition of oxygen rather than the cleavage of the hydrocarbon into smaller breakdown compounds, as first reported by Miller and Mayo in 1956 [31]. Yet, despite β-carotene’s highly unsaturated conjugated polyene backbone and the many β-carotene oxidation product studies carried out over several decades [32,33,34], it was not until 2014 that a β-carotene-oxygen copolymer product (“copolymer”) was reported by us as the main product of the spontaneous oxidation of β-carotene [8]. Prior to that, β-carotene autoxidation was known to produce only a mixture of many apocarotenoids. There appear to be no known previous reports of a copolymer product.

It is now known that β-carotene oxygen copolymers occur naturally [8,35,36] when β-carotene undergoes oxidative degradation in a wide range of plant products during storage or drying [35,36].

**Figure 1:**
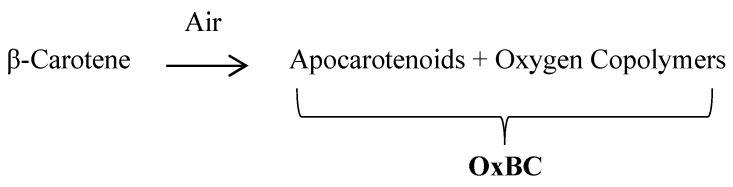
Formation of OxBC by spontaneous oxidation of β-carotene produces mainly a β-carotene oxygen copolymer compound and minor amounts of many apocarotenoid breakdown products.

In the laboratory, the full oxidation of β-carotene with air or pure oxygen in solution yields a complex, reproducible product, OxBC (Oxidized Beta-Carotene), containing 80–85% by weight of a copolymer compound and 15–20% of many small apocarotenoid breakdown products [8,37] (Figure 1).

**Figure 2:**
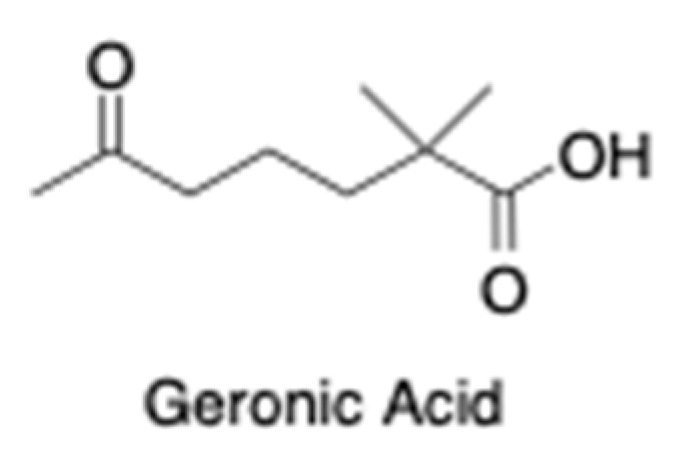
Geronic acid is an apocarotenoid product characteristic of OxBC that is used as a proxy marker compound for estimating OxBC.

The chemically complex OxBC is indirectly determined in foods and feeds using the apocarotenoid geronic acid (Figure 2) as a proxy marker compound for OxBC content [35]. 

**Figure 3:**
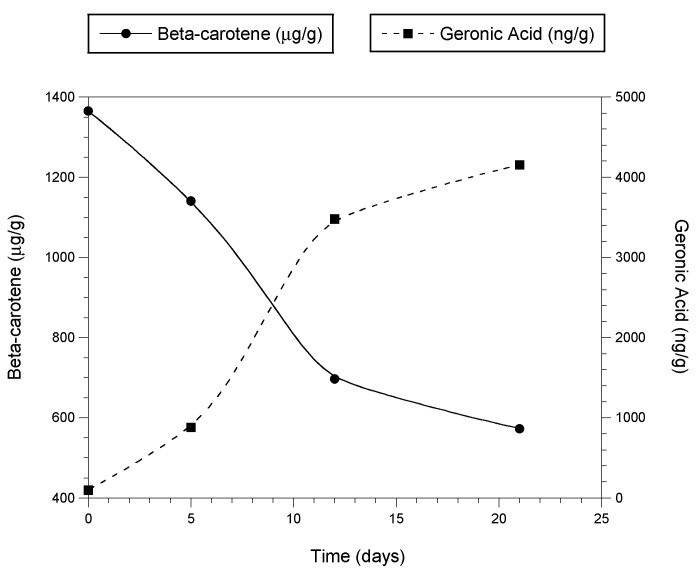
Formation of geronic acid as β-carotene is oxidized in dried, powdered carrot exposed to air. Measurements at time 0 used freshly dehydrated carrot purée. The dried purée was immediately ground to powder, spread thinly on a tray, and exposed to air and ambient light. Values at subsequent time points were determined using the powder.

Figure 3 illustrates the production of geronic acid as β-carotene is lost during the oxidation of dehydrated and powdered carrot over a period of days [38].

Synthetic OxBC is produced commercially by the full, non-enzymatic air oxidation of pure β-carotene in an ethyl acetate solution.

The term apocarotenoid is applied somewhat loosely here, not only to include the double bond cleavage products but also compounds derived from some of these products by further chemical transformations during the oxidation reaction. Some of these compounds are also termed norisoprenoids [39,40]. 

The OxBC apocarotenoids are formed as by-products of the polymerization reaction. In the reaction with air, the final polymer:apocarotenoid ratio is approximately 4:1 (*w*/*w*). OxBC’s apocarotenoids contain no more than 18 carbon atoms, less than half of β-carotene’s 40 carbons [8], with a few present at 1% levels and the rest at much lower levels. Notably absent are those apocarotenoids containing more than 18 carbons, including vitamin A. These more reactive longer-chain apocarotenoid products (≥C20) formed early in the reaction are ultimately consumed and removed by further oxidation. The copolymer compound has been shown to be partially susceptible to further breakdown into apocarotenoids under acidic and basic conditions [37]. 

Several of OxBC’s apocarotenoids possess flavor and fragrance characteristics [39], being present, for example, in leaf products (e.g., tobacco, tea, mate), many essential oils, fruits (grapes, passionfruit, starfruit, quince, apple, nectarine, tomato, melon), spices (saffron, red pepper), wine, rum, coffee, oak wood, honey, and seaweeds. Thirteen of OxBC’s identified apocarotenoids are Generally Recognized As Safe (GRAS) human flavor agents [41].

### 3.2. OxBC’s Dual Immunological Function

OxBC exerts actions on the immune system through pathways that are distinct from either vitamin A or intact β-carotene, neither of which are present in OxBC. Uniquely, OxBC exhibits dual immunological activities relating to (1) enhanced innate immune detection or priming and response to pathogens [9] and (2) anti-inflammatory/pro-resolution activity that mitigates excessive immune responses and reduces the level of background inflammation [42,43].

Several in vitro and in vivo studies have demonstrated the immune-modulating activities of OxBC [9,42].

An evaluation of the ability of OxBC to influence the expression of genes relevant to core immune responses using quantitative real-time PCR arrays showed a pattern of activity consistent with two key activities [8]. First, OxBC upregulated the expression of genes encoding products that function in pathogen sensing and the detection of pathogen-associated molecule patterns (PAMPs), including Toll-like receptors (TLRs) and other proteins that act as cofactors for PAMP detection, such as CD14 (clusters of differentiation 14) and lymphocyte antigen 92 (LY96/MD2). Second, OxBC also appeared to down-regulate the expression of genes associated with the initiation and propagation of inflammatory responses, suggesting anti-inflammatory potential. This effect was observed for inflammatory cytokines such as tumour necrosis factor (TNF) and interleukin-1β, but also for cytokine receptors and other molecules that promote an inflammatory reaction. Of particular interest was the finding that OxBC inhibited key signaling molecules and regulators of the NF𝜅B pathway. This pathway plays pivotal roles in signaling events initiated by both the TLR system and inflammatory mediators such as TNF, suggesting a potential common mechanism behind both patterns of gene expression. 

In vitro studies at the cellular level further clarified that it is the copolymers that are responsible for the innate immunological activities of OxBC, while the apocarotenoids appear to be inactive in this regard [9]. The results obtained for immune receptor levels, cytokine levels, and phagocytic activity indicated that OxBC could modulate innate immunity in a biologically beneficial way. These findings implied that the net result would be to prime the innate immune system to respond to subsequent challenges more rapidly. This is supported by the fact that OxBC had very little apparent effect on cytokine levels and phagocytic activity in the absence of a challenge. Unlike traditional immune stimulants, which directly trigger an inflammatory immune response, OxBC could potentially modulate but not stimulate and utilize an animal’s energy stores unless directly affected by stress.

As a result of the study of the biological activity of OxBC and other oxidized carotenoids, it has been proposed that carotenoid copolymer compounds are the actual agents responsible for many of the provitamin A-independent activities of β-carotene and other carotenoids [9].

It has not yet been established whether the source of anti-inflammatory activity of OxBC originates from the copolymer or the apocarotenoid fractions. In this regard it is of interest that β-ionone, a small molecule apocarotenoid present in OxBC, is reported to possess anti-inflammatory activity [44].

In addition to OxBC’s immune-modulating activity, several of OxBC’s apocarotenoids are recognized flavor agents [37,45], presenting the possibility that OxBC may also improve feed palatability.

### 3.3. Livestock Applications. Health and Performance and Metaphylaxis of Sub-Clinical Conditions

As described in more detail in Section 4, the utility of OxBC as a feed additive has been demonstrated in studies with piglets [46], sows [43], post-wean pigs [47], broiler chickens [48], and dairy cattle [42,49]. To illustrate, in piglets, dietary supplementation with OxBC improved growth performance and prevented the vaccine-induced growth lag associated with the PRRS (porcine reproductive and respiratory syndrome) vaccination [46]. In sows, supplementation with OxBC, beginning at late gestation and continuing through lactation, resulted in reduced proinflammatory cytokine levels in colostrum and milk concurrent with increased colostral and milk immunoglobulin levels [43]. In broilers, dietary supplementation with OxBC reduced the level of pathogen (Clostridium perfringens) recovered from the gut and protected against the reduction in growth performance associated with experimental induction of subclinical necrotic enteritis [48]. In dairy cattle, supplemental OxBC mitigated subclinical intramammary infections [49]. 

It has been proposed that the search for suitable alternatives to antibiotic growth promoters should yield substances that achieve effects similar to the antibiotics they are intended to replace, namely, a reduction in both bacterial load and inflammation [6,50,51,52]. The results from trials with pigs, poultry, and dairy cows highlight the utility of OxBC in achieving both outcomes. The benefits observed with piglets and gestating/lactating sows likely are consistent with the proposed anti-inflammatory actions of OxBC, whereas the reduction in C. perfringens in the poultry study and the elimination of the intramammary infections in lactating dairy cows support the benefits of innate immune priming. Note that OxBC has no direct anti-microbial effect and that the reduction or elimination of sub-clinical levels of bacterial pathogens in the poultry and dairy studies is consistent with the proposed immune priming actions, which positions host defenses to better detect and respond to the presence of various pathogens.

As of mid-2022, OxBC has been used commercially in the form of OxC-beta™ Livestock in various countries as a feed additive in approximately 16 million piglets, 94,000 sows, and 8.75 million poultry, and as a supplement for 12,000 dairy cows.

### 3.4. OxBC Safety

The question of the toxicity of β-carotene oxidation compounds arose indirectly as a result of the negative outcomes of several β-carotene human intervention clinical trials [53,54,55,56,57,58]. However, the physiological relevance of the cited supporting evidence, based entirely on in vitro model systems attempting to simulate oxidation conditions in vivo [59], was questioned in a review by an EFSA panel on the safety of β-carotene [60]. Additionally, synthetic OxBC does not contain any of the long-chain, retinoid-like apocarotenoids [8,60] that have been suggested as potentially toxic agents that may adversely interfere with vitamin A retinoid receptor activity. 

A recent toxicology study of OxBC in rats has established a wide safety margin for OxBC with a Maximum Tolerated Dose (MTD) of 5000 mg/kg, an LD_50_ of 30,079 mg/kg, and a No Observed Adverse Effect Level (NOAEL) of 1875 mg/kg body weight [45]. Synthetic OxBC has been supplemented in livestock, pets, and humans at approximately 0.5 mg/kg body weight/day, a level 3750-fold below the NOAEL-based threshold.

An OxBC uptake study in mice established that both OxBC polymer and associated apocarotenoid compounds were naturally present in all tissues and fluids examined [45].

The safety of OxBC has been formally evaluated by several national regulatory authorities around the world. Notably, New Zealand, Australia, Brazil, Mexico, Thailand, and Philippine authorities have approved OxBC as safe for use in feeds for all animal species. In addition, OxBC is approved as an active ingredient in veterinary health products for companion animals in Canada. Further support for the safety of synthetic OxBC is provided by the history of commercial use in animal feeds and health supplements in the above countries, spanning almost a decade, with no serious adverse events reported. Furthermore, the natural occurrence of OxBC in many commonly used animal and human feeds and foodstuffs [35,36] indicates a long history of exposure to dietary OxBC for both animals and humans.

### 3.5. OxBC Stability

OxBC, both alone and in the form of the OxC-beta™ Livestock feed additive (Avivagen Inc., Ottawa, ON, Canada), shows multi-year stability as established by extensive chemical testing over a period of several years and in accelerated aging studies. Activity is not affected by pelleting, for example.

## 4. OxBC as an Effective Alternative to Antimicrobials in Livestock Species

Clearly, a need has been established to identify and qualify natural immune modulators as replacements for antimicrobial drugs as a means to boost the immunity of commercial livestock and protect them from environmental pathogens [61]. The need has been made even more pressing by the rapid appearance of antimicrobial resistance associated with antimicrobials commonly used for swine, poultry, and other commercial species, and by consumer preference in the marketplace [62]. As described herein, the presumed mechanism of action of OxBC is to prime the innate immune system. This includes both pathogen recognition responses as well as the activation of anti-inflammatory mediators [9,42]. A series of studies were undertaken in poultry, swine, and cattle to test this hypothesis and to determine whether the effect of OxBC on the animal’s immune system would translate to better growth, productivity, and feed utilization. 

### 4.1. Poultry

Two initial studies were undertaken in broiler chickens at locations in Canada and the United Kingdom (UK) to determine the efficacy of OxBC in enhancing the growth of broilers [63]. In the Canadian trial, 0, 1, 2, or 5 ppm OxBC were added to the diet, whereas, in the UK trial, 0, 2, or 5 ppm OxBC were provided in a cornstarch or corncob grits premix to determine the optimum carrier for OxBC, as well as to monitor the effect of OxBC on growth and feed conversion. In the Canadian study, all levels of OxBC (1, 2, and 5 ppm) improved final body weights (BW) of the chickens compared with the unsupplemented and nonmedicated (no bacitracin methylene disalicylate (BMD) included) control birds after 39 days of feeding under typical Canadian commercial conditions. As well, all levels of OxBC improved feed conversion (FCR) during the finisher period, and 2 and 5 ppm OxBC improved the FCR relative to the negative control group over the full production cycle. OxBC had no effect on average daily feed intake (ADFI) or mortality, but 2 and 5 ppm OxBC increased broiler average daily gain (ADG). From this study in Canada, the optimal OxBC dose was determined to be 2 ppm, and this dosage has consistently been recommended to date to end users as beneficial for both practical and economic purposes. 

Interestingly, the birds in the medicated group in the Canadian trial did not gain significantly more weight than the negative, nonmedicated control birds. The AGP used in this trial, BMD, is known to target Gram-positive bacteria (e.g., *C. perfringens*) by interfering with protein synthesis and cell wall structure [64,65]. The inability of BMD to enhance growth in this study suggests that the birds may not have faced a particularly high level of pathogen-induced stress. However, OxBC did improve bird body weight and ADG relative to both the control and medicated groups, with the effects being most consistent at an inclusion rate of 2 ppm. Since OxBC has no direct antimicrobial activity, it was presumed that an anti-inflammatory mode of action was responsible for the positive effects on growth and performance that were observed in the birds treated with OxBC, especially given the lack of efficacy of BMD in the same trial. 

In the UK study, 2 or 5 ppm OxBC on cornstarch and 5 ppm OxBC on corncob grits improved all pertinent parameters (ADG, BW, and feed intake (FI)) when fed for the entire length of the study (35 days) and compared with the negative, nonmedicated control birds. However, feed conversion was not improved at any level of OxBC compared with the control group, and there were no differences between the three OxBC groups. When birds were fed 2 ppm OxBC on corncob grits, the overall ADG, BW, and FI were lower than the respective control group values, and bird mortality was higher than expected for all six experimental groups. It was concluded that cornstarch was the preferred carrier for OxBC, and, similar to the Canadian trial result, 2 ppm OxBC was determined to be the optimal dose, as there was no additional benefit evident when the dosage was increased to 5 ppm OxBC with the cornstarch carrier. 

An inconsistency between the trials was in the effect of OxBC on FCR. For the entire duration of the Canadian trial the birds fed the 5 ppm OxBC diet had the lowest FCR, and this was significantly lower than the FCR of the negative control and medicated groups. As well, all three OxBC groups registered a significantly lower FCR than did the negative control group, suggesting that OxBC can replace BMD. However, the medicated group birds had the lowest FCR during the grower period, and this was also significantly lower than the FCR for the 2 ppm OxBC birds. In an environment where the removal of AGPs from broiler diets is the ultimate objective, the most relevant comparison is OxBC versus no BMD, and in that case, the result was quite good. In the UK study, the birds that received OxBC had a lower FCR than the control birds during the starter and grower periods but not during the finisher or overall growth periods. The equivocal effect on FCR was explained to be likely the result of the increased FI that was evident in the UK trial rather than a negative effect on FCR. If there is a dual positive effect on weight gain and FI, the FCR will not necessarily change, as it is a ratio of the two measurements. 

Finally, the incidence of mortality during the two trials also differed. It was very low in the Canadian study, with no differences observed between the treatment groups. However, mortality was abnormally high in the UK study, although post-mortem necropsies ruled out any treatment-related causation for the birds. The low mortality during the Canadian trial provides further evidence, in addition to the lack of effect for BMD, that the stress on the birds was minimal, while the UK mortalities were attributed to locational, seasonal, or management differences. There were no differences in the mortality rates between the treatment groups in the UK study. 

In a study by Kang et al. [48], OxBC was tested as a replacement for commonly used AGPs (Virginiamycin-2 mg/kg and bacitracin-55 mg/kg) in Ross broiler chicks exposed to *Clostridium perfringens* sequelae to necrotic enteritis (NE). The experimental model called for a challenge dose of the pathogen to be administered to the chicks, followed by evaluation for clinical signs of infection and the monitoring of the birds’ growth performance. Changes in the integrity of the small intestine are directly related to NE, as evidenced by the development of lesions, so it was necessary to record lesion scores for all experimental groups throughout the course of the study to determine the efficacy of OxBC. In fact, the mean lesion scores of the OxBC treatment groups (2, 4, or 6 ppm) were significantly lower than that of the positive control group (*C. perfringens* challenge, no AGP or OxBC) and were similar to those found for the negative control (no challenge, AGP or OxBC) and antibiotic groups (Virginiamycin and bacitracin). This was highly significant in that reducing the intestinal lesion score in an infected bird increases its likelihood of recovering from NE and therefore being marketable. 

The mean body weights of the OxBC treatment groups also increased significantly compared to the positive control (*C. perfringens* challenge group). Although OxBC has no direct killing action on bacteria, it was noted that in conjunction with the improvement in lesion scores that could be attributed to OxBC, the number of clostridial bacteria in the intestine was also reduced in a dose-dependent manner. *C. perfringens* were not detected in the AGP groups at the end of the experiment, confirming the AGPs’ antimicrobial effect, while the residual values evident in the OxBC groups more closely resembled that found in the negative control group. It was further noted that the reduction in the intestinal load of *C. perfringens* was greater at the higher doses of OxBC (4 and 6 ppm) but decidedly less than was apparent in the positive control group by two orders of magnitude. Kang et al. questioned whether the more decided reduction in *C. perfringens* by the two AGPs was a desirable outcome, as the balance of the normal gut flora may have been unnecessarily disturbed by the AGPs. 

The AGPs proved to be better than OxBC at promoting growth in the face of the *C. perfringens* challenge, which is consistent with their action as growth promoters. However, it was noted that the positive control group displayed significant weight loss post-challenge, while the OxBC groups maintained body weight similar to that of the negative control group. Overall, Kang et al. concluded that the effects of OxBC were consistent with the innate immune priming activity of the compound that had been reported previously by Johnston et al. [9]. Furthermore, these researchers noted that OxBC in feed contributes to the prevention of necrotic enteritis in commercial broiler chickens and has a positive effect in improving the productivity of the birds. 

### 4.2. Swine

The earliest swine study with OxBC was conducted in Canada by Hurnik et al. [46]. The trial evaluated the effects of OxBC administered in feed to weaned barrows aged 18–21 days. The pigs were randomly and evenly distributed into two groups; one group was vaccinated with a commercial modified live viral vaccine against the porcine reproductive and respiratory syndrome (PRRS) virus, while the second group received a placebo. The two groups were then further divided into four treatment diet groups: 0 ppm (control), 10 ppm, 30 ppm, or 100 ppm OxBC, and the treatments began after an acclimatization period and continued for 28 days. 

The results from this study were the first indication of OxBC’s promise. The pigs displayed improved growth performance when they were given OxBC for 28 days, regardless of vaccine status. The 10-ppm group of pigs also grew faster (by ~12%), as determined by their ADG, than did pigs fed the control diet after being vaccinated with the modified live PRRS virus. In fact, the pigs that received 10 ppm OxBC were found to have the best FCR and ADG relative to the control and higher levels of OxBC for both the vaccinated and unvaccinated pigs. Hurnik et al. attributed this positive response to OxBC’s anti-inflammatory capacity, and they concluded that pigs treated with OxBC might be able to maintain better growth in the face of a viral challenge. The authors further stated that where it is safe and effective to use feed additives, OxBC can be useful as a non-antibiotic option in the husbandry of weaned pigs.

The Hurnik et al. study was followed by a more detailed and inclusive trial in Vietnam by Kinh et al. [47]. The objective of this study was to determine the effects of OxBC on the growth performance of pigs through an entire growth cycle under commercial production conditions in Vietnam. The study involved five dietary treatment groups with five replicate pens per treatment as follows: Control Basal diet: no antibiotics or OxBC; Basal diet: antibiotics (chlortetracycline, colistin sulfate), no OxBC; Basal diet: 2, 4 or 8 ppm OxBC, no antibiotics. The parameters measured were weight gain, FI, FCR, diarrhea incidence, and mortality. OxBC and antibiotics both improved growth rate, feed efficiency, and body weight compared to the unsupplemented control. However, the pigs that received 4 and 8 ppm OxBC also performed better than those that were treated with antibiotics, which was unexpected. During the starter phase (Days 1–28 post-wean), all three levels of OxBC reduced the occurrence of diarrhea in a dose-dependent manner and to a greater extent than the antibiotics.

OxBC addition to the feed of pigs at all three stages of the production cycle (Starter, Grower, Finisher) resulted in improved ADG and feed efficiency (G/F) and, thus, higher final body weights. The positive effects were most apparent during the Starter period, in comparison to the Grower and Finisher periods. This was not surprising given that the young pig is subjected to considerable stress in the form of weaning, diet change, handling, vaccination, environmental issues, and pathogen load at a time when the immune system is in the developmental stage. This was further evidenced by the highest incidence rate for diarrhea, which occurred during the Starter period, consistent with the susceptibility of young piglets to post-wean diarrhea [66]. Kinh et al. attributed this finding to the apparent immune-modulating action of OxBC. Of significance in this regard, Wu et al. [67] have reported in a study of the role of *E. coli* F18 infection in post-wean diarrhea in Chinese domestic piglets that the TLR signaling pathway and especially CD14 appear to play an important role in resistance to *E. coli* F18 infection.

The reduction in the incidence of diarrhea was proposed to explain the improved growth performance of piglets in the OxBC-supplemented pigs versus the Control group animals during the Starter period. As the animals aged, they would become more immunocompetent, and this would minimize the incidences of diarrhea during the Grower and Finisher phases, leading to improved growth performance. However, Kinh et al. suggested that improved feed palatability and increased ADFI may also have contributed to the improved growth of piglets in the OxBC groups during the Starter period. FI was stable across all treatments during the Grower period and decreased in the OxBC groups during the Finisher period, so if there was an FI effect, it was limited. It was suggested that rather than stimulating FI, OxBC had the post-wean effect of improving animal health and facilitating nutrient utilization as the trial moved past twenty-five days. This may have produced a nutrient (calorie)-sparing response, which is counterintuitive to what is normally assumed to happen to an animal’s feed intake in response to an immune challenge [68].

The Kinh et al. study demonstrated the benefits of OxBC feed inclusion on growth performance, feed efficiency, and piglet health during the post-wean period. The findings were particularly important for the commercial feed industry in Southeast Asia, given that the trial was conducted under typical Vietnamese commercial conditions. The data obtained from the trial support the use of OxBC to replace AGPs in swine feed, especially during the early production stages from post-wean through the first half of the Grower phase. Notably, in this trial, the performance and health benefits that were observed in groups fed OxBC were comparable to those of the AGP group. The conclusion of the researchers was that dietary supplementation with low parts-per-million levels of OxBC improves the productivity of pigs reared under Vietnamese commercial production conditions and supports the concept that OxBC will enhance swine growth and health in the absence of in-feed AGPs.

With OxBC firmly established as beneficial to production in pigs, the emphasis turned to breeding animals, with the assumption that improving the health of the sow would provide her piglets with a “head-start” in life by minimizing litter morbidity and mortality and improving sow and piglet health. Therefore, a study was undertaken at South China Agricultural University in Guangzhou, China [43] to evaluate the impact of dietary OxBC administration on sows during the perinatal period on immune status and productivity. Dietary OxBC supplementation at 4 and 8 ppm greatly enhanced colostrum IgM, IgA and IgG levels and the IgM and IgG content of 14-d milk. This was an important finding, as an increase in immunoglobulins in OxBC-treated sow colostrum and milk implies passive transference of the antibodies to the piglets and subsequent protection from the corresponding pathogens. Dietary OxBC supplementation decreased TNF-α and IL-8 levels in colostrum as well as TNF-α and IL-18 levels in 14-d milk. A tendency toward increased soluble CD14 (sCD14), an immune factor that plays an important role in the development of the immune system of the piglet, in colostrum and 14-d milk was also observed in OxBC-treated sows. Although dietary treatments did not significantly affect ADFI nor backfat thickness loss during lactation, OxBC appeared to enhance litter weight and individual piglet weight at weaning. 

The authors surmised that the evident reduction in pro-inflammatory cytokines observed in sow colostrum and milk was an indication that OxBC acted in an anti-inflammatory manner in the sows. They pointed out that perinatal sows often suffer from a metabolic syndrome which results in a low-grade systemic inflammatory condition [69]. The authors interpreted the response seen in sows as a consequence of improved mammary gland health.

The study conclusions also extended to the health performance of the farrowed piglets. Although not statistically significant, there was a trend toward higher litter and piglet weights at weaning for sows in the OxBC groups. Additional measurements suggested why these trends may have been real. The OxBC groups appeared to have higher levels of lactose in the 14-day milk relative to the control group.

### 4.3. Cattle

Two ruminant trials have been conducted to illustrate the effectiveness of OxBC in cattle. The first study, conducted in Canada, assessed the potential anti-inflammatory actions of OxBC in calves under an experimental model of bovine respiratory disease (BRD) [42].

The initial phase of the study included the in vitro treatment of primary bovine neutrophils with OxBC induced cell-selective, caspase-3–dependent apoptosis. Conversely, the OxBC treatment did not induce neutrophil necrosis. Neutrophil apoptosis and their subsequent clearance by macrophages are key mechanisms in the resolution of inflammation, whereas neutrophil necrosis is implicated in the propagation of excessive inflammatory activity. Thus, the initial findings suggested the possibility that OxBC could represent a novel nutraceutical and non-antibiotic approach to reducing the severity of inflammation associated with bovine respiratory disease (BRD). This possibility was next assessed in an in vivo challenge study in Holstein calves. 

The calves were first given a dietary supplement of OxBC for 28 days and then inoculated with *Mannheimia haemolytica*. The bronchoalveolar lavage fluid was collected at 3 and 24 h post-challenge and analyzed for markers of apoptosis. Dietary OxBC enhanced leukocyte apoptosis in bronchoalveolar lavage fluid as well as subsequent efferocytosis by macrophages without altering the numbers of circulating leukocytes. These findings confirmed that OxBC could confer anti-inflammatory benefits in cattle with respiratory tract disease.

The second study with OxBC in ruminants was conducted in New Zealand [49]. The focus was on the all-too-common intramammary infection of dairy cows, which results in increased somatic cell counts (SCC) as manifested in the cow and in the bulk tank milk. The accompanying increased risk of clinical mastitis and the culling of cows have a significant negative economic impact on the dairy farm [70]. The intent of the study was to determine whether OxBC could be an effective agent for bacteriological cure and in preventing clinical mastitis and increased SCC in cows with subclinical intramammary infections.

Pasture-fed cows with confirmed intramammary infection (quarter-level SCC >200,000 cells/mL and a recognized bacterial intramammary pathogen) were enrolled from four dairy herds. The animals were randomly assigned to control or OxBC treatment groups and were individually fed from Day 0 for an average of 40 days with 0.5 kg of a cereal-based supplementary feed that either contained 300 mg of OxBC (treatment; *n* = 129 quarters) or did not (control; *n* = 135 quarters). Samples of milk were collected on Days 21 and 42 for microbiology and SCC analysis.

The bacteriological cure rate of 13.9% was greater for quarters of cows in the OxBC treatment group than for cows in the control group (6.9%). For clinical mastitis, its incidence on Day 42 was lower in cows from the treatment group (only one out of 129 cows, or 0.78%) than in cows from the control group (six out of 135 cows, or 4.44%). Mean quarter-level SCC did not differ between the groups. Together, the results indicated that when the cows were fed 300 mg OxBC/day, a higher bacteriological cure rate was realized, as was a lower prevalence of intramammary infection and a lower incidence of clinical mastitis compared to untreated controls. Increased bacteriological cure rate, the reduced prevalence of intramammary infection, and clinical mastitis are key outcomes that could ultimately lead to reduced reliance on the therapeutic use of antibiotics to treat clinical mastitis in the dairy industry. 

## 5. Conclusions

Oxidized β-carotene (OxBC) is a phytochemical that spontaneously forms when β-carotene reacts with oxygen in the air. It is characterized by a predominance of β-carotene–oxygen copolymer compounds. Copolymer-rich, synthetic OxBC, obtained by the full oxidation of β-carotene in a solvent, exhibits unique, dual-acting immunological activities by (a) priming the innate immune system and (b) limiting the propagation of excessive inflammation. The innate immune priming action is effected by the activity of the copolymer product. The molecular origin of the anti-inflammatory effects has not yet been elucidated.

Immunological effects are achieved via mechanisms that are distinct from the provitamin A or antioxidant pathways commonly proposed as explanations for carotenoid actions.

Livestock trials with low, parts-per-million level supplementation of synthetic OxBC in feed have shown performance benefits over and above the benefits of feeds containing regular vitamin and mineral premixes. The benefits observed with piglets and gestating/lactating sows are consistent with the anti-inflammatory actions of OxBC, whereas the reduction in *C. perfringens* in the poultry study and the elimination of the intramammary infections in lactating dairy cows support the benefits of innate immune priming. OxBC has no direct antimicrobial effect, and the metaphylactic reduction or elimination of bacterial pathogens in the poultry and dairy studies reflects actions on the host’s immune system, which is better able to detect and respond to the presence of pathogens. 

The global feed industry is characterized by very tight profit margins making product application cost a critical consideration in the development and adoption of viable AGP alternatives. Synthetic OxBC of high purity and quality is readily and economically produced on a commercial scale. Current and increasing use by multiple repeat customers in the poultry, swine and dairy sectors in various countries attests to the economic viability of OxBC as a feed additive.

OxBC, through its ability to enhance immune function, health, and performance, has demonstrated utility not only as a viable alternative to in-feed antibiotics but also is able to provide tangible health and performance benefits in applications where antibiotic usage is precluded (e.g., milk production).

The introduction of synthetic vitamin A replaced forages formerly used as a source of provitamin A, with the result that β-carotene and its oxidation products have largely disappeared from many livestock feeds. Supplementing feeds with synthetic OxBC can restore the previously unrecognized additional benefits of β-carotene and its oxidation products.

The natural origins, wide safety margin, host-mediated mode of action, and the demonstrated efficacy of OxBC are important characteristics to be considered in the development of products aimed at improving the sustainability of modern livestock practices. 

## Data Availability

Supporting data are provided in the relevant cited references.
